# The Expression of SIRT1 and DBC1 in Laryngeal and Hypopharyngeal Carcinomas

**DOI:** 10.1371/journal.pone.0066975

**Published:** 2013-06-21

**Authors:** Xue-Min Yu, Ying Liu, Tong Jin, Jun Liu, Juan Wang, Chao Ma, Xin-Liang Pan

**Affiliations:** 1 Department of Otolaryngology, Qilu Hospital of Shandong University, Jinan, China; 2 Department of Otolaryngology Head and Neck Surgery, West China Hospital, Sichuan University, Chengdu, China; University of Saarland Medical School, Germany

## Abstract

**Rationale and Objective:**

Sirtuin 1 (SIRT1) plays an important role in tumorigenesis and is increased in many human tumors. DBC1 is a negative regulator of SIRT1 via promotion of p53-mediated apoptosis. It is necessary to investigate the expression of SIRT1 and DBC1 in laryngeal and hypopharyngeal squamous cell carcinomas (LSCC and HSCC) and its correlation with available clinical parameters.

**Methods:**

The mRNA levels of SIRT1 and DBC1 were measured in 54 paired LSCC or HSCC tumors and corresponding adjacent noncancerous mucosae using quantitative RT-PCR (qRT-PCR). The protein levels of SIRT1 and DBC1 were also evaluated in 120 cases of patients with LSCC or HSCC using immunohistochemical staining. The correlation between SIRT1 and DBC1 expression and clinical parameters was analyzed with Pearson chi-square test.

**Results:**

qRT-PCR assay showed that, compared with the paired adjacent noncancerous mucosae, SIRT1 mRNA was significantly decreased in tumors. The immunohistochemical results indicated that the SIRT1 protein was also downregulated in tumors compared with noncancerous mucosae. Moreover, decreased SIRT1 was significantly correlated with the tumor clinical stage and lymph node metastasis. Additionally, DBC1 mRNA was significantly increased in tumors compared with noncancerous mucosae. The immunohistochemical results indicated that the DBC1 protein was downregulated in tumors, which is inconsistent with the results obtained by qRT-PCR. Finally, decreased DBC1 protein was significantly correlated with tumor differentiation, lymph node metastasis, and p53 expression.

**Conclusions:**

SIRT1 and DBC1 might be involved in the pathophysiology of laryngeal and hypopharyngeal squamous cell carcinomas and are associated with lymph node metastasis and p53 positive staining in LSCCs and HSCCs.

## Introduction

Head and Neck Squamous Cell Carcinoma (HNSCC) is the sixth most malignancy in the world [1]. Approximately, one-fourth of all HNSCCs are laryngeal squamous cell carcinoma (LSCC) [2]. In contrast, hypopharyngeal squamous cell carcinoma (HSCC) is not so common, but it is usually diagnosed in the advanced stage with poor diagnosis [3]. Although several treatment strategies, including surgery, radiotherapy, gene therapy and immunotherapy, have been developed for LSCC and HSCC, no treatment could achieve a satisfactory therapeutic outcome for patients and the survival rate has not been improved significantly [4]. Therefore, identification of prognostic markers will be important for the prevention and therapy of LSCC and HSCC.

Sirtuin 1 (SIRT1), which is nicotinamide adenine dinucleotide (NAD+)-dependent deacetylase, belongs to the silent information regulator 2 (Sir2) family of sirtuin histone deacetylases (HDACs) [5], [6]. SIRT1 participates in energy metabolism, genomic stability [7], placental cell survival [8], and neuroprotection [9], and it may play an important role in tumorigenesis. It is reported that SIRT1 is increased in many human tumors, such as breast cancer [10], ovarian cancer [11], prostate cancer [12], gastric cancer [13], colon cancer [14], diffuse large B-cell lymphoma [15], acute myeloid leukemia [16] and Bowen’s disease [17]. However, there is also a great deal of evidence showed that the expression of SIRT1 in breast cancer, ovarian carcinoma, bladder carcinoma, prostate carcinoma, and glioblastoma, is lower than that in normal tissues [18]. SIRT1 can be activated by SRT, AROS and SUMO-1 and can be inhibited by tenovins, DBC1 and Tat [19].

DBC1 is well known as a negative regulator of SIRT1. Previous studies revealed that DBC1 promotes p53-mediated apoptosis through specific inhibition of SIRT1 [20], [21]. Therefore, DBC1 has been suggested as a tumor suppressor based on these evidence. However, some recent studies showed that DBC1 is much more overexpressed in breast cancers [10] and colorectal cancers [22] relative to normal tissues [23]. Therefore, it is still difficult to determine whether DBC1 is a tumor suppressor or a tumor promotor.

In the present study, we aim to elucidate whether SIRT1 and DBC1 are involved in the development of LSCCs and HSCCs and to identify the potential role of SIRT1 and DBC1 in the prevention and therapy of LSCC and HSCC in the clinic.

## Materials and Methods

### Patients and Sample Collection

A total of 120 malignant tumors and 54 adjacent noncancerous tissue specimens (mucosae) from patients having undergone surgical treatment for primary HSCC or LSCC at Qilu Hospital of Shandong University (Jinan, China) from January 2009 to April 2011, were included in the current study. Those patients who had received neoadjuvant chemotherapy or radiation therapy before surgery were excluded from this study. Fifty-nine of the patients were diagnosed LSCC and 61 patients were diagnosed HSCC. Fresh specimens were divided into two sections; one section was preserved with TRIzol reagent (invitrogen, USA) for gene expression profiling and the other section was fixed in formalin for histological evaluation. The TNM classification was in accordance with the International Union against Cancer (UICC, 2002) TNM Staging. Approval for this study and the consent procedure was obtained from the Institutional Review Board of Shandong University (No. 12040). All patients in this study were provided written informed consent to participate in advance.

### RNA Extraction and Quantitative Real-Time PCR

Total RNA was isolated from the tissues using TRIzol reagent (invitrogen, USA) and then was reverse-transcribed to cDNA using PrimeScript Reverse Transcriptase (Takara, Dalian, China) following the manufacturer’s protocol. Quantitative real-time PCR was performed using the SYBR Green chemistry in the ABI 7900HT Sequence Detection (ABI Applied Biosystems, Foster City, CA). The gene-specific primers used were as follows [23], [24]: SIRT1 (forward primer 5′-TGG CAA AGG AGC AGA TTA GTA GG-3′, reverse primer 5′-CTG CCA CAA GAA CTA GAG GAT AAG A-3′), DBC1 (forward primer 5'-ATG TCC CAG TTT AAG CGC CAG-3', reverse primer 5'-CAA CCC CAA AGT AGT CAT GCA A-3'); and GAPDH (forward primer 5'-CCT GTT CGA CAG TCA GCC G-3', reverse primer 5'-CGA CCA AAT CCG TTG ACT CC-3').

PCR reaction was performed in a 10 µl reaction volume which consisted of an initial denaturation step at 95°C for 30 sec and following amplification by 40 cycles at 95°C for 5 sec, 60°C for 30 sec. The threshold cycle (Ct) is defined as the cycle number at which the fluorescence passed a pre-determined threshold. For expression analysis, the experiment was designed to use the matched non-tumor tissue as the control, and the expression level of SIRT1 and DBC1 in tumor tissue was calculated by using the 2^−ΔΔCT^ relative quantification method [25] with GAPDH as a reference. Both target and reference (GAPDH) genes were amplified in separate wells in triplicate.

### Immunohistochemistry

Immunostaining for SIRT1 and DBC1 was carried out in either malignant tumors or adjacent noncancerous mucosae. Fresh specimens were embedded in paraffin and sectioned at 4 mm with a Leica microtome (Leica, Wetzlar, Germany). Following deparaffination and rehydration, the tissue sections were treated with a microwave antigen retrieval procedure in sodium citrate buffer for 12 min. Then sections were treated with 3% H_2_O_2_ for 15 min to quench endogenous peroxidase activity and blocked with goat serum (10%) for 20 min. Slides stained with primary antibodies and negative controls were incubated at 4°C overnight. Tissue sections without primary antibody addition served as a negative control. Then the slides were incubated with the secondary antibody at room temperature for 30 minutes. DAB (3, 3-diaminobenzidine) was used as a substrate for color development. All slides were counterstained with hematoxylin. The following primary antibodies were used: anti-SIRT1 (1∶200, ab32441, Abcam, Cambridge, UK), anti-DBC1 (1∶200, ab84384, Abcam, Cambridge, UK), and anti-p53 (1∶400, DO-1, Santa Cruz Biotechnology, Santa Cruz, CA).

### Evaluation of IHC Staining and Scoring

The expression levels of SIRT1 and DBC1 were scored by multiplying the staining intensity by the immunopositive cells that were stained. The semi-quantification for immunostaining intensity was scored according to the following scale: 0, no staining; 1, mild staining; 2, moderate staining; and 3, marked staining. Average numbers of immunopositive cells within the samples were determined in at least five areas at × 400 magnification. The percentage of immunopositive cells was scored on a scale of 0 (0–9%), 1 (10–29%), 2 (30–49%), 3 (50–74%) and 4 (≥75%) [11], [26], [27]. The maximum combined score was 12 and the minimum combined score was zero. So the immunohistochemical expression of SIRT1 and DBC1 was defined according to the following scores; negative, combined score 0; low expression, combined score 1–8; high expression, combined score 9–12. As to p53, it was regarded as positive if more than 10% of the tumor cells showed immunoreactivity [11]. The immunohistochemical analysis was performed by two pathologists (MC and LCW) with consensus without knowledge of the clinicopathological information.

### Statistical Analysis

All data were analyzed with SPSS statistical software, version 14.0 (SPSS, Inc, Chicago, IL). To compare the mRNA expression levels between tumor tissues and adjacent noncancerous mucosae, the Wilcoxon matched-pairs signed-ranked test was used. The Spearman rank analysis was used to analyze the correlation between the mRNA levels of SIRT1 and DBC1. Mann-Whiney 2-tailed test was performed to compare the protein expressions of SIRT1 and DBC1 in tumor tissues and adjacent noncancerous mucosae. And the association between staining index and other categorical factors potentially predictive of prognosis was analyzed using Pearson chi-square test. In all analyses, values were considered significant at a *P* value of less than 0.05.

## Results

### Patient Characteristics

The characteristics of patients participated in this experiment are shown in **Table 1** **and** **Table 2**. The patients were grouped according to their age; sex; tobacco exposure; alcohol consumption; differentiation; clinical stage (I and II versus III and IV); presence of lymph node metastasis; and p53 staining results.

**Table 1:** Clinicopathologic variables and the protein expression status of SIRT1.

			SIRT1 expression n (%)			
Characteristics	No. of cases (%)		Negative	Low	High	p
Age (years)	<60	67 (56)	18 (27)	30 (45)	19 (28)	0.769
	≥60	53 (44)	12 (22)	23 (43)	18 (34)	
Sex	Female	11 (9)	3 (27)	3 (27)	5 (45)	0.439
	Male	109 (91)	27 (25)	50 (46)	32 (29)	
Tobacco	<10/day	22 (18)	7 (31)	7 (31)	8 (36)	0.428
	≥20/day for more than 10 years	98 (82)	23 (23)	46 (47)	29 (30)	
Alcohol	<200 ml/day	47 (39)	14 (29)	17 (36)	16 (34)	0.353
	≥200 ml/day for more than 10 years	73 (61)	16 (22)	36 (49)	21 (29)	
Differentiation	Well	25 (21)	7 (28)	11 (44)	7 (28)	0.518
	Moderate	51 (42)	16 (31)	20 (39)	15 (29)	
	Poor	44 (37)	7 (16)	22 (50)	15 (34)	
TNM stage	I+II	42 (35)	17 (40)	18 (42)	7 (17)	**0.005**
	III+IV	78 (65)	13 (17)	35 (45)	30 (38)	
LN metastasis	No	68 (57)	20 (29)	34 (50)	14 (21)	**0.021**
	Yes	52 (43)	10 (19)	19 (37)	23 (45)	
p53	Negative	64 (53)	16 (25)	27 (42)	21 (33)	0.862
	Positive	56 (47)	14 (25)	26 (46)	16 (28)	

The *P* values were obtained by Pearson’s chi-square analysis. SIRT: Sirtuin; LN: lymph node.

**Table 2:** Clinicopathologic variables and the protein expression status of DBC1.

			DBC1 expression n (%)			
Characteristics	No. of cases (%)		Negative	Low	High	p
Age (years)	<60	67 (56)	2 (3)	34 (51)	31 (46)	0.761
	≥60	53 (44)	2 (4)	30 (56)	21 (39)	
Sex	Female	11 (9)	0	3 (27)	8 (73)	0.113
	Male	109 (91)	4 (4)	61 (56)	44 (40)	
Tobacco	<10/day	22 (18)	1 (4)	9 (40)	12 (54)	0.432
	≥20/day for more than 10 years	98 (82)	3 (3)	55 (56)	40 (41)	
Alcohol	<200 ml/day	47 (39)	2 (4)	23 (48)	22 (46)	0.707
	≥200 ml/day for more than 10 years	73 (61)	2 (3)	41 (56)	30 (41)	
Differentiation	Well	25 (21)	0	16 (64)	9 (36)	**0.026**
	Moderate	51 (42)	3 (6)	32 (63)	16 (31)	
	Poor	44 (37)	1 (2)	16 (36)	27 (61)	
TNM stage	I+II	42 (35)	1 (2)	24 (57)	17 (40)	0.789
	III+IV	78 (65)	3 (4)	40 (51)	35 (45)	
LN metastasis	No	68 (57)	1 (1)	44 (65)	23 (34)	**0.013**
	Yes	52 (43)	3 (6)	20 (39)	29 (56)	
p53	Negative	64 (53)	2 (3)	26 (41)	36 (56)	**0.009**
	Positive	56 (47)	2 (4)	38 (68)	16 (28)	

The *P* values were obtained by Pearson’s chi-square analysis. DBC1: deleted in breast cancer 1; LN, lymph node.

The age of the patients included in this study varied from 38.0 to 81.0 years, with a mean ± SE of 58.1±1.0. Most of the patients (91%) are males and 82% of the patients are smokers. Lymph node metastasis occurred in 43% (52/120) of them. Among the 120 patients, 65% (78/120) of the cases were at late stage (III and IV). The percentage of well, moderate and poor differentiation are 21% (25/120), 43% (51/120) and 37% (44/120), respectively. p53 immunostaining was positive in 47% (56/120) of these patients.

### Quantitative RT-PCR Analysis for the Expression of SIRT1 and DBC1 in Carcinomas (LSCCs and HSCCs) and Noncancerous Mucosae

**Figure 1:**
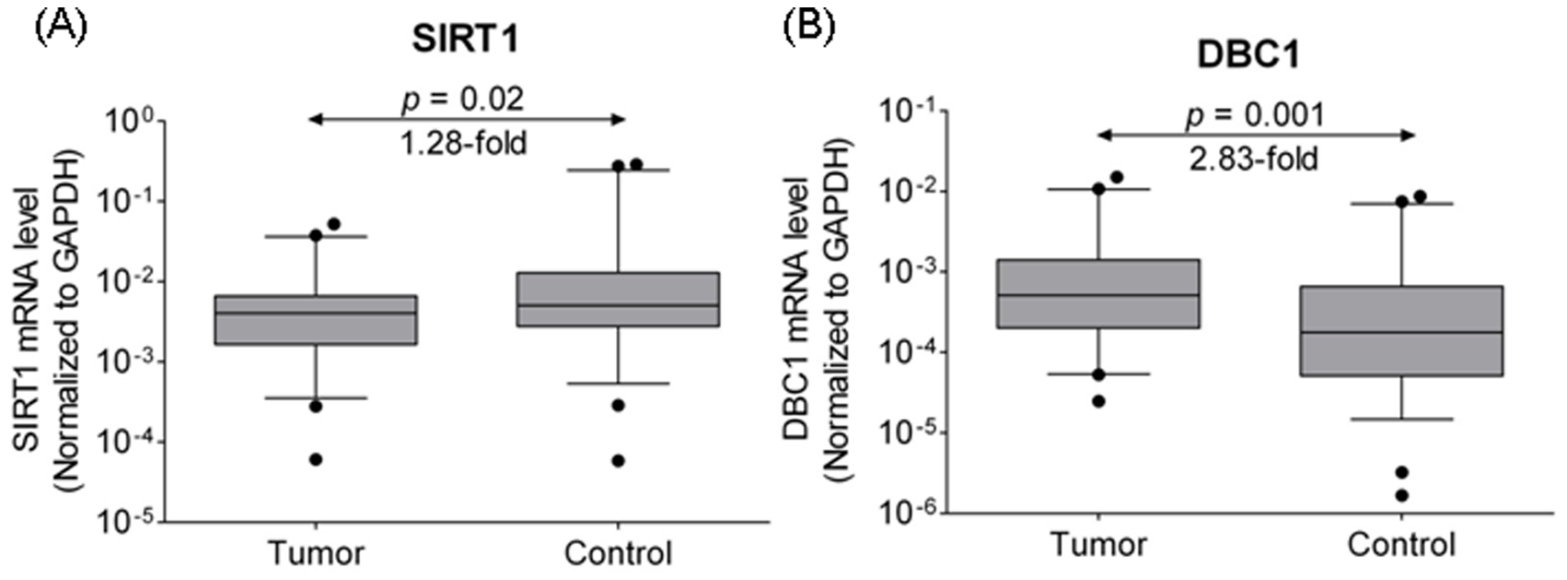
Quantitative determination of mRNA by means of real-time RT-PCR for SIRT1 (A) and DBC1 (B) in tissues from laryngeal and hypopharyngeal carcinomas or adjacent noncancerous mucosae. Target mRNA relative expression levels were given as ratios of GAPDH transcript levels in the same RNA sample. Box plots were shown with medians plus 5th to 95th percentile whiskers. Statistical analyses were performed with the Wilcoxon matched paired test (2-tailed).

**Figure 2:**
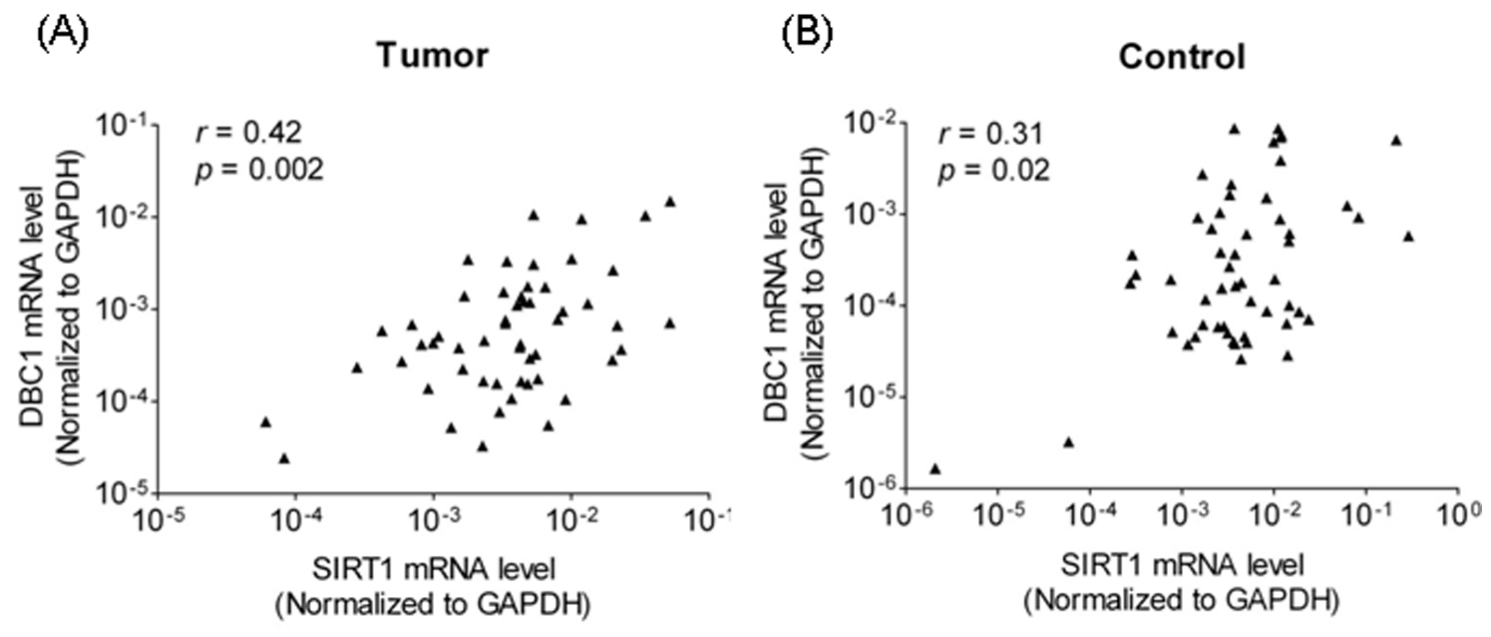
Relationship between mRNA levels of SIRT1 versus DBC1 in tumors (laryngeal and hypo pharyngeal carcinomas) (A) and adjacent noncancerous mucosae (B). The correlation coefficient (r) was calculated by using Spearman rank analysis. *P* values were considered significant at less than 0.05. (*n*  = 54).

Quantitative RT-PCR was performed to confirm SIRT1 and DBC1 mRNA expression in 54 pairs of carcinomas (LSCCs and HSCCs) and adjacent noncancerous mucosae. The relative expression levels of target mRNA were given as ratios of GAPDH transcript levels in the same RNA sample. It was found that the SIRT1 mRNA expression decreased significantly in carcinomas compared with adjacent noncancerous mucosae (*P*  = 0.02, 1.28-fold, Fig. 1A). The DBC1 mRNA expression was significantly higher in carcinomas than in adjacent noncancerous mucosae (*P*  = 0.001, 2.83-fold, Fig.1B). In addition, the Pearson chi-square test showed there is a trend of the mRNA expression of SIRT1 and DBC1 was correlated with each other both in carcinomas (*r*  = 0.42 Fig. 2A) and in adjacent noncancerous mucosae (*r*  = 0.31, Fig. 2B). However, the *r* value is too low to identify the relationship between DBC1 and SIRT1 expression. Further different population and larger number of participants are needed.

### Immunohistochemical Analysis for the Expression of SIRT1 and DBC1 in Carcinomas (LSCCs and HSCCs) and Noncancerous Mucosae

**Figure 3:**
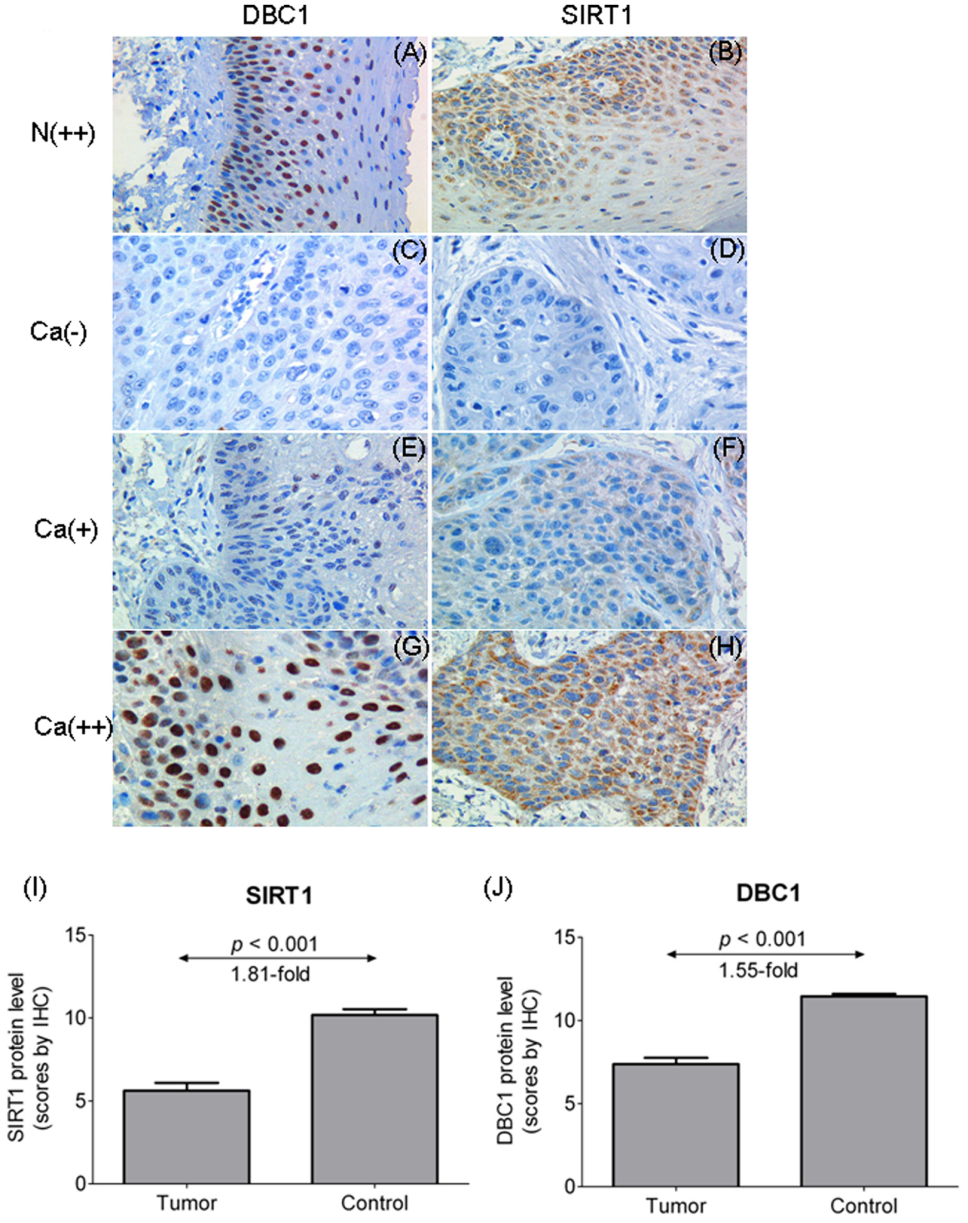
Expression of DBC1 and SIRT1 protein determined by means of immunohistochemistry in noncancerous mucosae (A, B) and the carcinomas (C–H). N, noncancerous mucosa; Ca, laryngeal or hypopharyngeal carcinoma; (−), negative immunostaining; (+), positive (low expression); (++), positive (high expression). Original magnification × 400. Expression of SIRT1 (I) and DBC1 (J) protein was determined by means of immunohistochemistry in carcinomas or adjacent noncancerous mucosae. Statistical analyses were performed with the Mann-Whiney 2-tailed test. All *P* values were considered significant at less than 0.05.

**Figure 4:**
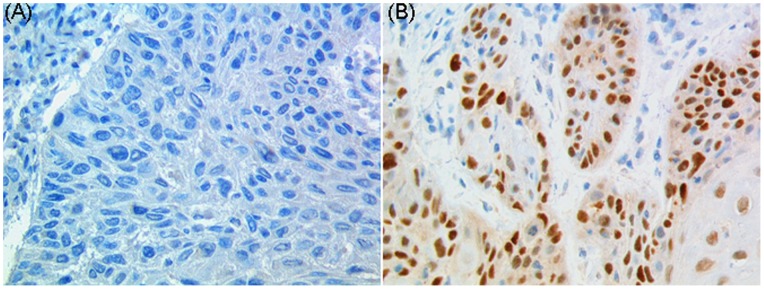
Expression of p53 protein determined by means of immunohistochemistry in laryngeal or hypopharyngeal carcinoma. (A), negative staining; (B), positive staining. Original magnification × 400.

Immunohistochemical assay was performed to investigate the protein expression of SIRT1 and DBC1 in 120 carcinomas (LSCCs and HSCCs) and 54 adjacent noncancerous mucosae. High expression of SIRT1 was observed in 31% (37/120) of tumors and 61% (34/54) of adjacent noncancerous mucosae. And high expression of DBC1 was observed in 43% (52/120) of tumors and 93% (50/54) of adjacent noncancerous mucosae. Immunoreactivity for DBC1 was found primarily in the nuclei (Fig. 3A, C, E, G). SIRT1 was expressed in the nuclei and cytoplasm, mainly in the cytoplasm (Fig. 3B, D, F, H). With statistical analysis (Mann-Whiney 2-tailed test), it was found that SIRT1 protein expression was significantly decreased in carcinomas than in adjacent noncancerous mucosae (Fig. 3I, *P*<0.001, 1.81-fold). Inconsistent with the results obtained by quantitative real-time PCR, DBC1 protein expression was found to be significantly lower (Fig. 3J, *P*<0.001, 1.55-fold) in carcinomas than in adjacent noncancerous mucosae. The IHC staining of p53 is shown in Fig. 4A, B, which shows that p53 was expressed primarily in the nuclei.

### Association of SIRT1 and DBC1 Protein Expression with Clinicopathologic Characteristics of Patients

**Figure 5:**
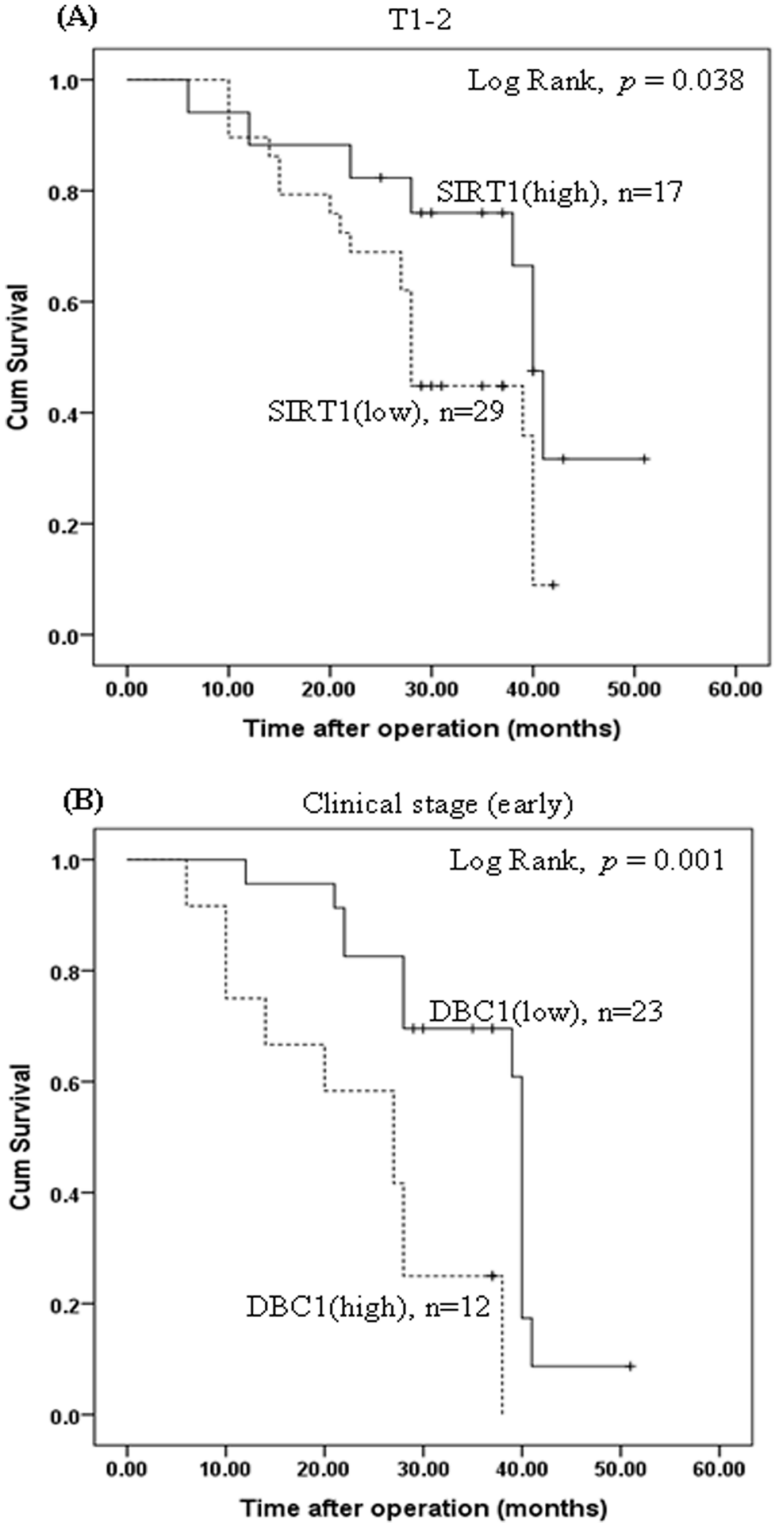
The level of SIRT1 and DBC1 are correlated with patient survival. (A) the survival analysis in low and high level of SIRT1 protein expression; (B) the survival analysis in low and high level of DBC1 protein expression. The survival analysis was performed using Log Rank test.

At protein level, the expression of SIRT1 was significantly correlated with the tumor clinical stage (*P*  = 0.005) and lymph node metastasis (*P*  = 0.021). The expression of DBC1 protein was significantly correlated with tumor differentiation (*P*  = 0.026), lymph node metastasis (*P*  = 0.013), and p53 expression (*P*  = 0.009). Other variables showed no statistically significant association with the expression of SIRT1 and DBC1 in LSCCs and HSCCs (Table 1 and 2). Furthermore, there are 120 malignant tumors patients were included in the current study, of which 59 cases were LSCC, 61cases were HSCC. At the end of the follow-up, 9 cases dropped off, 58 patients died, 53 cases survived. Moreover, we performed the survival analysis using Log Rank test and found that SIRT1 (Fig. 5A) and DBC1 (Fig. 5B) are correlated with patient survival.

## Discussion

Laryngeal squamous cell carcinoma is one of the most common cancers worldwide. About 40,000 new cases of LSCC are estimated each year in Europe and half of the cases will ultimately die due to the disease [28]. Squamous cell carcinoma from hypopharynx (HSCC) accounts for 5% of all head and neck cancers and almost 50% of patients recurred mostly within the first year [3]. At present, the effective treatment for LSCC and HSCC is surgical removal, along with chemotherapy and/or radiotherapy. However, the prognosis for LSCC and HSCC remains poor because about 40% of patients were announced with advanced tumors at the first diagnosis [29]. Some novel biomarkers can direct us to learn the change of cells during disease process, which should be useful for predicting the prognosis and designing individual therapy for the patients with carcinomas [30]. Ideal biomarkers can offer early diagnosis for the patients with LSCC and HSCC, particularly for the early-stage carcinomas. In recent years, the identification of ideal cancer biomarkers has become the major focus of cancer research [30], [31].

In this study, we evaluated the expression of SIRT1 and DBC1 in LSCCs and HSCCs at both mRNA level and protein level. Associations between clinicopathological variables and the expression of SIRT1 and DBC1 in cancer tissues were investigated. The main findings are that: (i) SIRT1 was down-regulated in tumor tissues compared with adjacent noncancerous mucosae at both mRNA level and protein level; (ii) DBC1 was overexpressed in tumor tissues at mRNA level but not at protein level; (iii) SIRT1 downregulation in tumor tissue was significantly associated with lymph node metastasis, and TNM stage; (iv) Downregulation of DBC1 in tumor tissue at protein level is significantly correlated with lymph node metastasis, tumor differentiation, and p53 expression; (v) SIRT1 is positively correlated with DBC1 in both carcinomas and adjacent noncancerous mucosae at mRNA level.

The current findings demonstrates for the first time that SIRT1 was down-regulated in LSCCs and HSCCs compared with the adjacent noncancerous mucosae, which was certificated by quantitative real-time PCR and immunohistochemistry. In agreement with our findings, decreased expression of SIRT1 has been reported in several tumors such as glioblastoma, bladder carcinoma, prostate carcinoma, and various forms of ovarian cancers [18]. In this study, down-regulation of SIRT1 is correlated with certain unfavorable prognostic factors including lymph node metastasis and late clinical (TNM) stage, indicating that SIRT1 may be involved in lymph node metastasis and the development of LSCCs and HSCCs, which seems to provide some valuable information for the judgement of prognosis and choice of therapies for clinicians. SIRT1 was initially considered to be an exclusively nuclear protein, while cytoplasmic staining of SIRT1 was recognized in a small number of cancers [32]. However, it has been reported previously that SIRT1 was mainly localized in the nucleus of normal cells in human tissues, but predominantly localized in the cytoplasm of cancerous [33]. Moreover, other researchers have revealed that SIRT1 is highly expressed in the cytoplasm of colon cancer cell lines and increased in colon tumors compared to normal colon tissues [34]. Therefore, we need further studies to clarify whether SIRT1 produces the same function with localization in nucleus and cytoplasm.

DBC1, a negative regulator of SIRT1, was known as an independent prognostic factor in gastric cancer, which correlates with a shorter overall survival and significantly associates with lymph node metastasis, advanced TNM stage, and tumor invasion [13]. In the present study, the expression of DBC1 is firstly described in the human LSCCs and HSCCs. The mRNA level of DBC1 was up-regulated in LSCCs and HSCCs compared with the adjacent noncancerous mucosae. However, the protein expression of DBC1 in tumor tissues is lower than that in adjacent noncancerous mucosae. The reasons for this result may include experimental and theoretical factors. Unlike quantitative real-time PCR, immunohistochemistry is a semi-quantitative experiment, so there must be some deviations that cannot be eliminated although we have 120 cases included in this study. And this is one of the limitations for this study. As we all know, gene expression is usually regulated at several stages, including transcription, post-transcription, and post-translational modification [19]. The translational and post-translational regulations can cause the difference between mRNA and protein expression [35]. And the mechanism is very complex, so we need more experiments to find out the exact reason. Downregulation of DBC1 in tumor tissue at protein level correlates with lymph node metastasis, poor differentiation of tumors and p53 immunoreactivity. Further work is needed to determine the precise role of DBC1 in tumorigenesis of LSCCs and HSCCs.

The mRNA expression of SIRT1 and DBC1 was positively correlated with each other both in carcinomas and in adjacent noncancerous mucosae, but the protein expression of them had no correlation with each other (data not shown). So in LSCCs and HSCCs, SIRT1 may be negatively regulated by DBC1 at transcriptional level. The interaction between SIRT1 and DBC1 and its effect on carcinogenesis in specific tumorigenic environments need further investigation.

It has been evidenced that DBC1 is a negative regulator of SIRT1 and promotes p53-mediated apoptosis through specific inhibition of SIRT1 [20], [21]. Our current data revealed that DBC1 protein was significantly lower in carcinomas compared to noncancerous mucosae, suggesting that decreased DBC1 might be involved in the development of laryngeal carcinoma tissues. Moreover, SIRT1 could attenuate apoptotic signals by deacetylating the DNA repair factor Ku70, which has recently been shown to suppress apoptosis by removing the pro-apoptotic factor Bax from the mitochondria in the cytoplasm [36]. We also found that downregulation of DBC1 protein in tumor tissue correlates with p53 immunoreactivity. Therefore, it is necessary in the future study to investigate the effects of DBC1 and SIRT1 on pro-apoptotic factors and signal pathways related to the induction of apoptosis, especially in the epithelial tumors. Furthermore, experiments to determine the effect of the expression of SIRT1 and DBC1 on cellular biological behavior in vitro are also necessary to understand the function of SIRT1 in LSCCs and HSCCs.

In conclusion, the results suggest that SIRT1 is downregulated and works as a tumor suppressor in LSCCs and HSCCs. However, it is still difficult to define the exact role of DBC1 in the development of LSCCs and HSCCs. A further prospective study is needed to determine the value of SIRT1 and DBC1 as candidate biomarkers and also to evaluate the usefulness as a survival factor for LSCCs and HSCCs. The findings of our study provide the preconditions for a further study of the mechanism related to carcinogenesis in LSCCs and HSCCs.
